# Case Report: Free-Floating Intracoronary Thrombus: Who Is the Convict?

**DOI:** 10.3389/fonc.2022.825711

**Published:** 2022-03-08

**Authors:** Francesca Mantovani, Ambra Paolini, Andrea Barbieri, Giuseppe Boriani

**Affiliations:** ^1^ Dipartimento di Medicine Specialistiche, Azienda USL-IRCCS di Reggio Emilia, Reggio Emilia, Italy; ^2^ Department of Medical and Surgical Sciences, Haematology Unit, Policlinico Hospital, Modena and Reggio Emilia University, Modena, Italy; ^3^ Department of Cardiology, Policlinico Hospital, Modena and Reggio Emilia University, Modena, Italy

**Keywords:** myocardial infarction, hypercoagulable state, myeloproliferative neoplasm, risk factors, coronary thrombus

## Abstract

In young patients, especially with no traditional coronary risk factors, hypercoagulable states may always be considered as an alternative cause of acute coronary syndromes. The concomitant thrombotic and bleeding risk associated with myeloproliferative disorders complicates the decision-making, particularly regarding long-term dual antiplatelet therapy. The chosen therapy may need to be frequently revisited, depending on the patient’s bleeding complications. We reported the case of a 49-year-old woman with acute myocardial infarction with no traditional risk factors for coronary artery disease where a myeloproliferative neoplasm was diagnosed.

## Clinical Case

**Figure 1:**
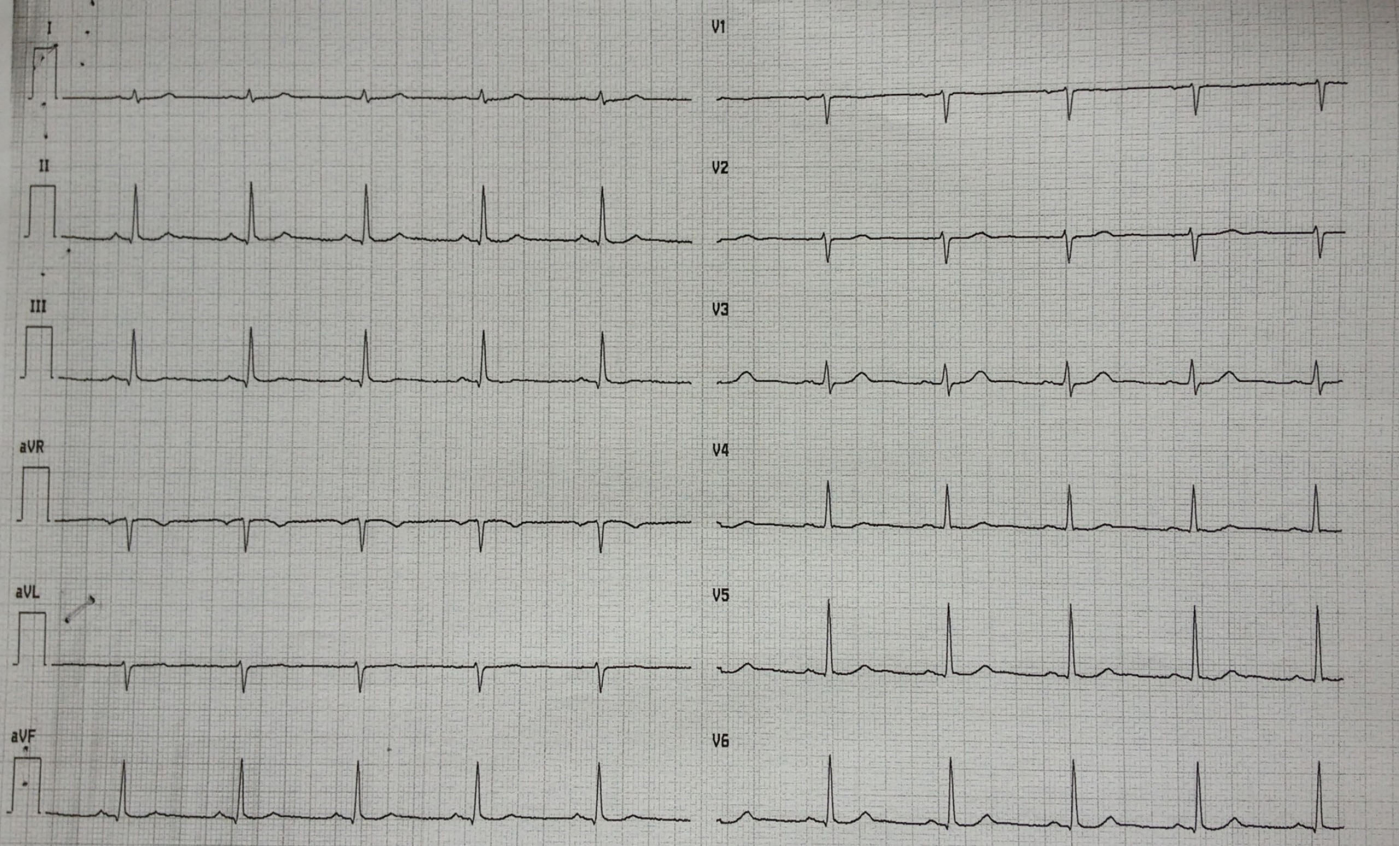
ECG at clinical presentation.

A 49-year-old woman presented to the emergency department with ongoing chest pain that started 4 h earlier. The12-lead electrocardiogram was unremarkable (**Figure 1**). Previously, the patient was hospitalized twice for non-ST-elevation myocardial infarction with a demonstration of normal coronary angiography in both cases. She was discharged in aspirin regimen but she has spontaneously withdrawn the drug due to the increased bleeding of her usual meno-metrorrhagia (due to uterine fibromatosis). She denied alcohol or illicit drug abuse, but she had a severe smoking habit. On physical examination, she was afebrile; the blood pressure was 110/85 mmHg, the heart rate was 65 beats per minute, and the oxygen saturation was 98% while she was breathing ambient air. Cardiac examination revealed a regular rhythm without extra heart sounds, no jugular venous distention, and no lower-extremity edema. Breath sounds were normal in both lungs. No hepatic and spleen enlargement were found. The first cardiac troponin I level was 0.13 ng/ml (normal value, <0.06), subsequently showing a peak of 12.25 ng/ml. The white cell count was 11,900 per cubic millimeter (n.r. 4,000–10,000 per cubic millimeter) with a normal differential count. The platelet count was high: 766,000 per cubic millimeter (n.r. 150,000–1,450,000 per cubic millimeter). The hemoglobin level was 11.1 g/dl with a mild decrease in red blood cell mean corpuscular volume (MCV 81 fl, n.r. 82–98 fl). Iron deficiency status was documented as follows: ferritin < 10 ng/ml (n.r. 37–150 ng/ml), serum iron 20 mcg/dl (n.r. 37–150 mcg/dl), and transferrin total iron biding capacity 558 mcgFe/dl (n.r. 275–500 mcgFe/dl). The international normalized ratio was 0.99, and partial thromboplastin time was 0.86 s (normal range, 0.82 to 1.24 s). The level of low-density lipoprotein cholesterol was 166 mg/dl, that of high-density lipoprotein cholesterol was 48 mg/dl, and that of triglycerides was 50 mg/dl. Results of other blood chemical and liver function tests were unremarkable.

**Figure 2:**
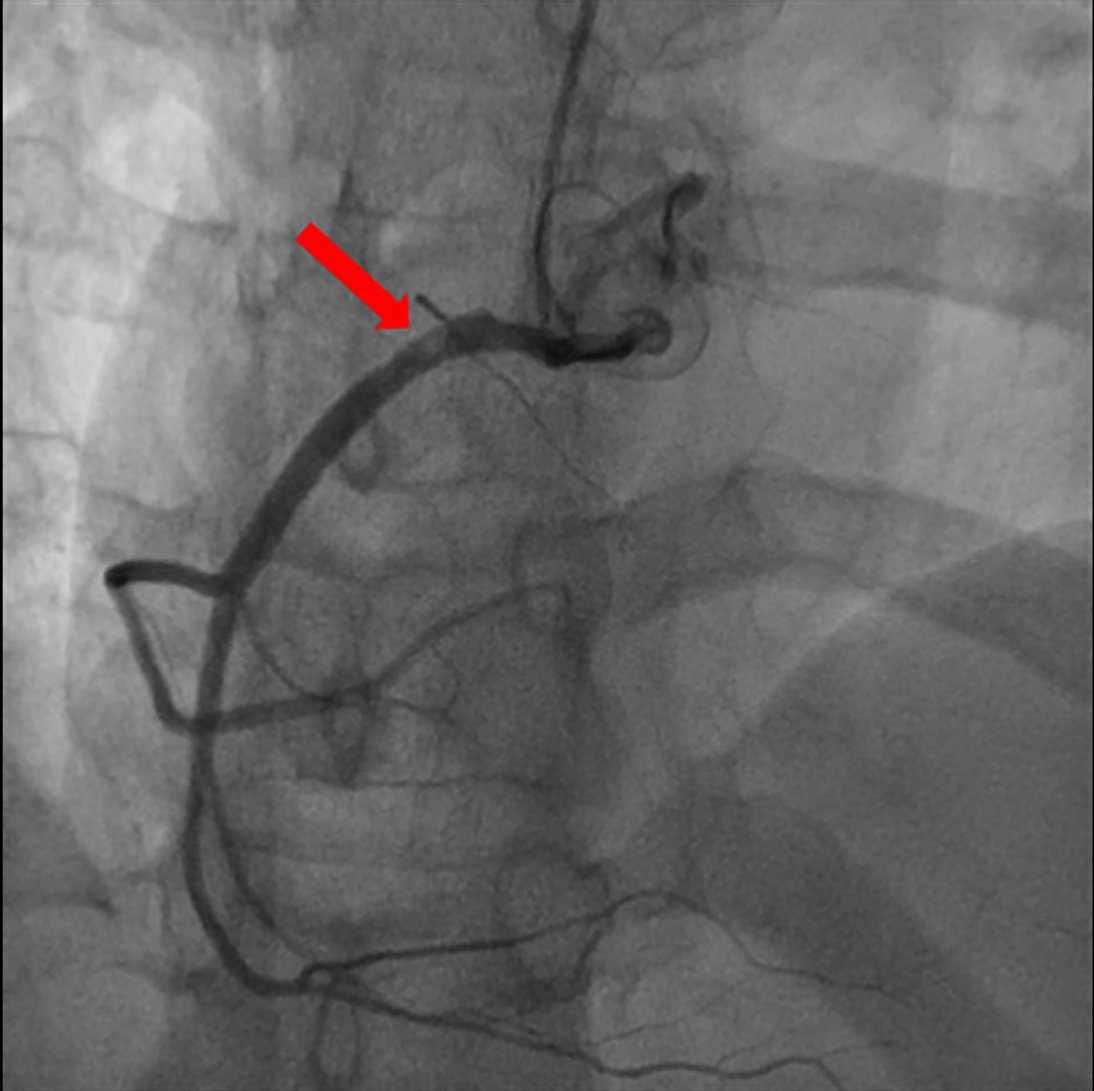
Coronary angiography: non-obstructive free-floating intracoronary thrombus of the right coronary artery (red arrow).

**Figure 3:**
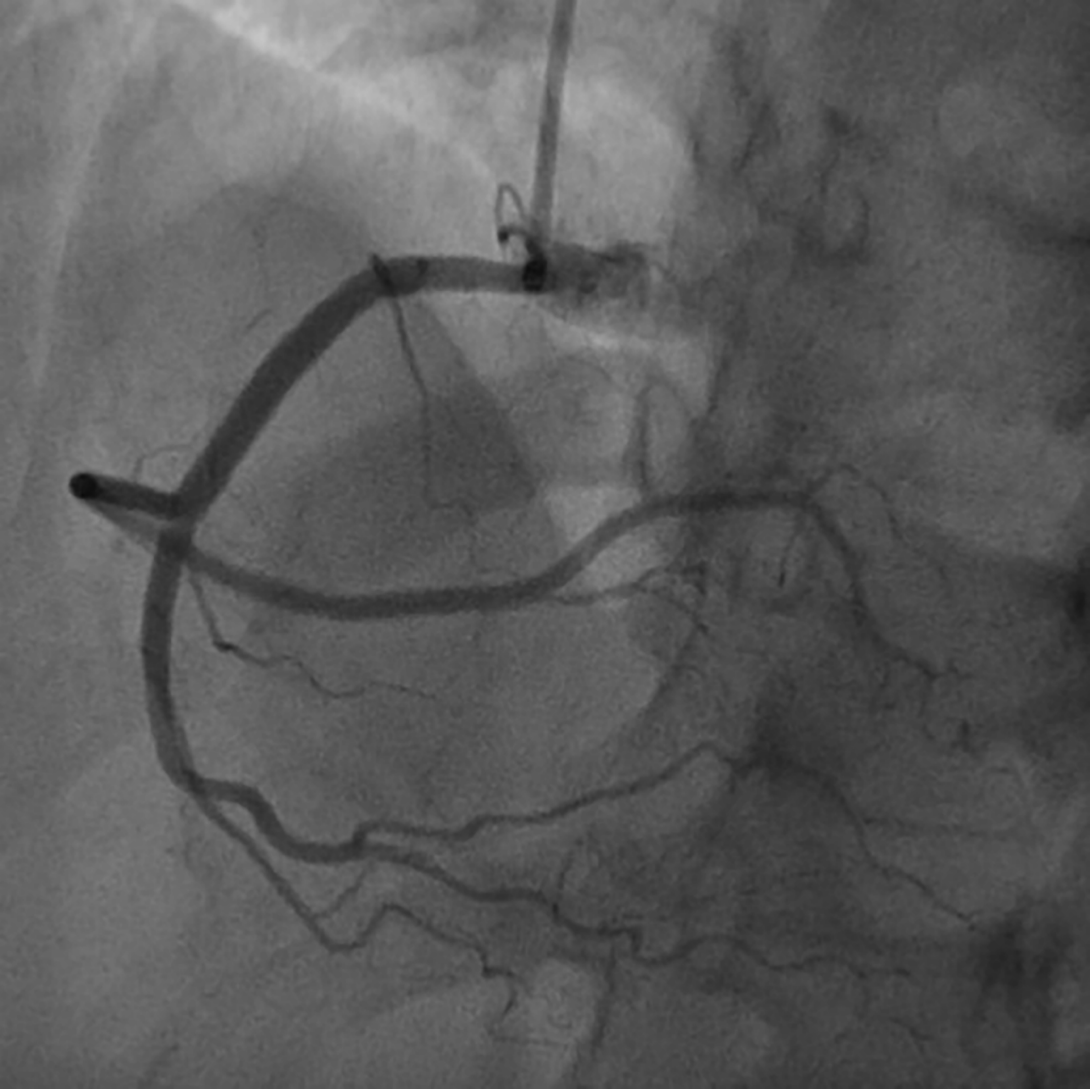
Coronary angiography with complete resolution of the thrombus formerly observed.

On the basis of chest pain and troponin elevation, a new non-ST-elevation myocardial infarction was diagnosed and standard therapy for acute coronary syndrome was instituted, i.e., aspirin (500 mg i.v.), atorvastatin (40 mg orally), clopidogrel (300 mg orally), and enoxaparin (4000 U i.v.). Continuous infusion of nitroglycerin was started. The patient underwent urgent coronary angiography, showing a non-obstructive free-floating intracoronary thrombus of the right coronary artery (**Figure 2**). The left main, left circumflex, anterior descending artery, and the rest of the left coronary arteries did not have angiographic signs of coronary atherosclerosis. The patient was treated with tirofiban: an initial infusion of 50 mcg/ml at 32 ml/h given in 30 min (total 800 mcg in 30 min) followed by a continuous infusion of 8 ml/h (400 mcg/h). After 24 h therapy, the patient underwent a second coronary angiography, which showed the complete resolution of the thrombus formerly observed (**Figure 3**).

**Figure 4:**
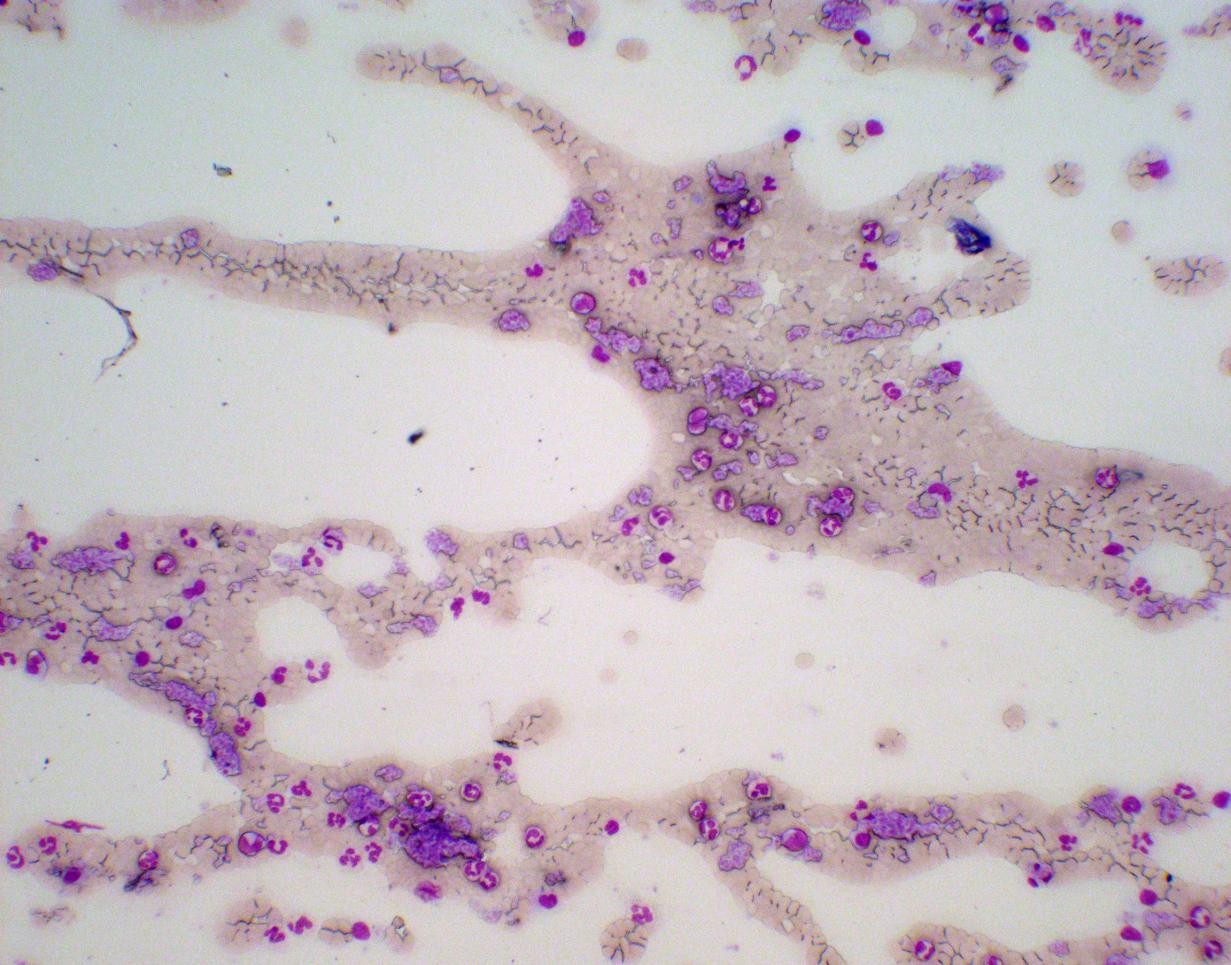
Panoramic screenshot of the peripheral blood (PB) smear “tail” on May-Grunwald-Giemsa (MGG) staining (100× magnification). Platelet aggregates, neutrophil granulocytes, lymphocytes, and monocytes (normal cells of the peripheral blood) of PB smear on MGG staining (400× magnification).

The following day, the white cell count was 9,020 per cubic millimeter, the hemoglobin level was 11.2 g/dl, and the platelet count was 602,000 per cubic millimeter. The patient recalled being informed about a high platelet count—approximately 500,000 per cubic millimeter—when she was 18 years old, after a routine blood exam, but the finding was not investigated further at that time. Even though iron deficiency anemia may be associated with thrombocytosis, given the persistent higher-than-expected cell counts, the possibility of a myeloproliferative neoplasm (MPN) with an associated secondary thrombophilia was considered (1). Levels of protein C and protein S were normal. Antithrombin III activity was 114% of the normal range, which is 80% to 120%. Tests for the prothrombin gene and V Leiden factor mutation were negative. Levels of anticardiolipin and anti-β2-glycoprotein antibodies were normal. A peripheral blood (PB) smear showed thrombocytosis, without relevant erythrocyte morphological alterations or leukoerythroblastic features (**Figure 4**). Leukocyte alkaline phosphatase score was moderately increased.

PB molecular screening detected the presence of the Janus Kinase 2 (JAK2) V617F mutation, whereas the Breakpoint Cluster Region-Abelson (BCR-ABL)-1 fusion transcript, pathognomonic for chronic myeloid leukemia, was absent (2). Bone marrow biopsy was not performed early after cardiac event due to bleeding risk on both anticoagulant and antiplatelet treatment (3). According to available data, an operative diagnosis of BCR-ABL-1 negative MPN, more suggestive for essential thrombocythemia, was formulated and, according to contemporary risk stratification algorithm (high-risk ET for thrombosis history and JAK2 mutation) (4), cytoreductive treatment with low-dose hydroxyurea was started.

At the 1-month follow-up visit, she was symptoms-free, with no further angina episodes. Her platelet count decreased to 448,000 per cubic millimeter. At 6 months’ and 1, 2, and 3 years’ follow-up, the patient was still symptom free, with no further thrombotic episodes, and no hemorrhagic complications. At the last hematologic follow-up as an outpatient, she was still tolerant and responsive to cytoreductive treatment.

Written informed consent was obtained from the patient for the publication of any potentially identifiable images or data included in this article.

## Discussion

When a myocardial infarction occurs in young patients without traditional risk factors for coronary artery disease, alternative causes should be sought. Important causes include hypercoagulable states, coronary vasospasm, coronary inflammation, anomalous coronary arteries, coronary dissection, and embolization (5).

In the present case, the patient’s persistent thrombocytosis was highly suspicious, suggesting the presence of an MPN. This group of myeloproliferative disorders is known to be associated with both hypercoagulability (6) and bleeding (7). Multiple mechanisms may account for these blood diatheses: hyperviscosity, platelet-aggregation abnormalities, leukocytosis leading to increased activation of the coagulation system, and various downstream effects of the *JAK2* mutation (8, 9). The concomitant thrombotic and bleeding risk complicates the decision-making, particularly regarding long-term dual antiplatelet therapy. However, current risk stratification of newly diagnosed patients with chronic BCR-ABL-1 negative MPN is primarily designed to estimate the likelihood of thrombotic complication (10). For this reason, we decided for long-term low-dose aspirin and clopidogrel therapy in order to reduce the risk of recurrent events, although this regimen may need to be frequently revisited, depending on the patient’s bleeding complications.

In one of the largest retrospective analysis of patients with polycythemia vera and essential thrombocythemia, the incidence of recurrent thrombosis after the first episode was 7.6% patient-years and age older than 60 was the only independent predictor of events. Of note, in the subgroup of patients with a previous acute coronary syndrome, a crucial role in reducing the risk of recurrence (70% reduction in the risk) was played by the use of cytoreductive drug together with antiplatelet agent (11). Current treatment is still mainly focused on reduction of thrombosis risk, control of myeloproliferation, improvement of symptom burden, and management of disorder-associated complications (12, 13).

## Conclusion

Hypercoagulable states may be an uncommon cause of acute myocardial infarction, even in the presence of traditional coronary risk factors. In these cases, clinical management may be particularly challenging due to contemporary high thrombotic and bleeding risk.

## Data Availability Statement

The original contributions presented in the study are included in the article/supplementary material. Further inquiries can be directed to the corresponding author.

## Ethics Statement

Written informed consent was obtained from the patient for the publication of any potentially identifiable images or data included in this article.

## Author Contributions

All authors contributed to the draft, and reviewed and approved the final version of the present case report.

## Conflict of Interest

The authors declare that the research was conducted in the absence of any commercial or financial relationships that could be construed as a potential conflict of interest.

## Publisher’s Note

All claims expressed in this article are solely those of the authors and do not necessarily represent those of their affiliated organizations, or those of the publisher, the editors and the reviewers. Any product that may be evaluated in this article, or claim that may be made by its manufacturer, is not guaranteed or endorsed by the publisher.
